# Chromosome-level genome assembly of *Trichomonas vaginalis* strain IR-78 (ATCC 50138)

**DOI:** 10.1038/s41597-025-06182-3

**Published:** 2025-11-05

**Authors:** Mostafa Y. Abdel-Glil, Johannes Solle, Heinrich Neubauer, Lisa D. Sprague

**Affiliations:** 1 Friedrich-Loeffler-Institut, Institut für Bakterielle Infektionen und Zoonosen (IBIZ), Naumburger Str. 96a, 07743 Jena, Germany; 2 Jena University Hospital – Friedrich Schiller University, Institute for Infectious Diseases and Infection Control, Jena, Germany

**Keywords:** Comparative genomics, Parasite genetics

## Abstract

*Trichomonas* vaginalis is a flagellated protozoan parasite and the causative agent of trichomoniasis, the most prevalent non-viral sexually transmitted infection, with significant impact on public health and economy. Here, we present a chromosome-level genome of *T. vaginalis* strain IR-78 (ATCC 50138), assembled using short and long-read sequencing technologies as well as chromatin conformation capture sequencing (Hi-C). The assembled genome is 173.3 Mb in size, with an N50 scaffold length of 26.4 Mb. The assembly is anchored in six super-scaffolds, corresponding to the six chromosomes of *T. vaginalis*, covering 93.5% of the genome. A total of 43,326 protein-coding genes were predicted, of which 95.4% were annotated using multiple databases, including Gene Ontology, eggNOG, and KEGG. Among these, 16,656 genes encoded hypothetical proteins. This high-quality genome assembly provides an additional resource for ongoing research on *T. vaginalis* pathogenesis, drug resistance, and host-parasite interactions. It furthermore enables comparative genomic studies to assess genomic variety across different *Trichomonas* species.

## Background & Summary

*Trichomonas vaginalis* is a protozoan parasite that causes trichomoniasis, the most common non-viral sexually transmitted infection (STI), affecting approximately 156 million people worldwide annually (WHO, 2025)^1^. This parasite mainly infects the urogenital tract and symptoms range from mild irritation to severe inflammation. However, a considerable proportion of infections are asymptomatic, which contributes to its widespread transmission^2,3^. *T. vaginalis* can cause serious health complications in women if left untreated, with complications including infertility, premature birth, pelvic inflammatory disease, cervical cancer and increased vulnerability to HIV. In men, the infection is frequently asymptomatic, prostatitis and urethritis may occur^1^. The public health implications of *T. vaginalis* are particularly severe in low- and middle-income countries in which access to healthcare and programmes for preventing sexually transmitted diseases is limited.

The true global burden of *T. vaginalis* is underestimated for numerous reasons among which are: inadequate understanding of its epidemiology and public health implications, asymptomatic carriers, inadequate diagnostic capabilities in low-resource settings and because *T. vaginalis* infection is not a notifiable disease^4,5^. Efforts to contain its spread face challenges, such as the lack of routine screening in many healthcare systems, the stigma associated with sexually transmitted diseases, and the parasite’s ability to persist in asymptomatic carriers. The problem is exacerbated by the emergence of drug-resistant *T. vaginalis* strains, which complicates treatment and prolongs transmission and infection cycles. Addressing these issues requires improved diagnostics, routine screening programmes and the development of new therapeutic options to fight resistance^4^.

The genome of *T. vaginalis* is characterised by its size and complexity^6^; it is approximately 160 Mb in size and consists to a significant extent of repetitive sequences and transposable elements. This genomic diversity may play a role in the parasite’s ability to evade the host’s immune system and to develop drug resistance^7^. However, only a limited number of *T. vaginalis* genome sequences are currently available in public repositories. Genome sequencing of further strains will enable comparative analyses and could reveal strain-specific patterns of pathogenicity.

**Fig. 1:**
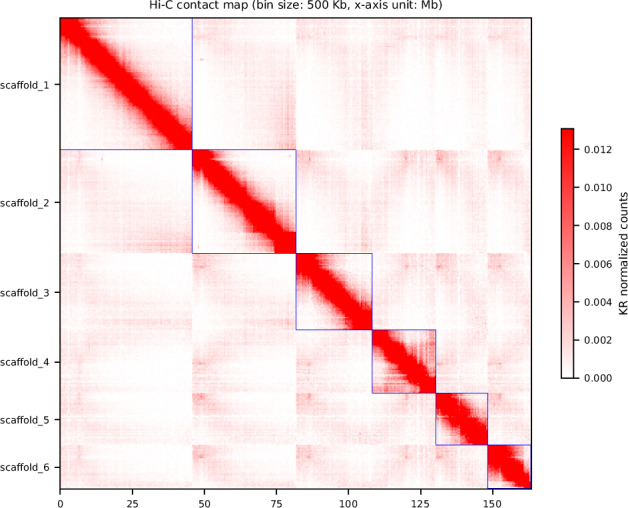
Hi-C contact map of the *Trichomonas vaginalis* IR-78 genome assembly. Chromosomal scaffolds are depicted. Interaction frequencies of Hi-C data are indicated by the color gradient bar on the right.

This work describes the construction of a chromosome-level genome assembly for *T. vaginalis* IR-78 (ATCC 50138) using Illumina short-read, Nanopore long-read, and Hi-C sequencing techniques. The final genome assembly of strain IR-78 is 173.3 Mb in size with a N50 scaffold length of 26.4 Mb (Fig. 1, Table 1). The six super-scaffolds, representing the genome’s six chromosomes, account for 162 Mb of the total genome size. The analysis revealed 43,326 predicted protein-coding genes.

**Table 1:** Genome statistics of *Trichomonas vaginalis* strain IR-78.

Metric	Value
Total length of assembly	173,3 Mb
Total length of chromosomes (Mb)	162,17 Mb (93,5%)
Total number of contigs	204
G + C content (%)	32,79%
Average coverage (with long reads)	30,2-fold
N50 scaffold/contig length	26,4/1,13 Mb
n. protein-coding genes	43326
Average gene length (bp)	1204
n. tRNA genes	460

## Methods

### *T. vaginalis* IR-78 sequencing

Genomic DNA was extracted from *T. vaginalis* IR-78 cultured in InPouch® TF-Bovine (Megacor, Austria) at a concentration of 1.5 × 10⁶ cells/mL. The extraction process utilized UNSET lysis buffer, followed by phenol-chloroform purification as described by Horner *et al*. (1996). DNA quality was evaluated using a NanoDrop Spectrophotometer (Thermo Fisher, USA), while quantification was performed with a Qubit Fluorometer (Invitrogen, USA).

Long-read sequencing was conducted with the R9.4.1 chemistry from Oxford Nanopore Technologies (ONT) on a GridION platform, using the SQK-LSK109 ligation kit. Data acquisition and basecalling were conducted via Guppy 6.5.7 using the sup-accurate model, yielding 3.65 million reads totalling 5.21 billion bases. Trimming and filtering of these ONT reads were performed with Porechop (default mode) and Filtlong (-min_length 1000), producing a refined dataset of 850,194 reads with an average length of 4.7 kb and a cumulative base count of 4 billion bases.

Short-read sequencing was carried out on an Illumina MiSeq platform using the XT DNA Library kit, generating 300 bp paired-end reads. This resulted in 52.7 million raw reads, corresponding to 13.9 billion bases. Adapter trimming and quality control using fastp v0.23.2^8^ improved the data quality, reducing the dataset to 51.4 million high-quality reads with a total of 13.2 billion bases and a Q30 score of 86.3%.

### *De novo* genome assembly

The genome assembly was performed using a hybrid approach based on long-read assembly with subsequent polishing using short reads to enhance both contiguity and accuracy. The assembly utilized the tricho-workflow pipeline (available at https://gitlab.com/FLI_Bioinfo/tricho-workflow, accessed on 12.11.2024), which has previously been successfully applied to *Tritrichomonas foetus*.

Nanopore long reads, filtered to include only those with a minimum length of 1000 bases, were used for the initial assembly with Flye v2.9^9^. The draft assembly underwent multiple rounds of polishing. Two iterations of Racon v1.5.0^10^ with Minimap2 v2.24^11^ were applied to refine the assembly using the long-read data. This was followed by two additional polishing rounds with Polypolish with bwa v0.50^12^ using high-quality, fastp-processed Illumina reads. Illumina data corrected 116,679 substitution errors in the draft assembly, achieving a final consensus quality of 99.87%. Nanopore reads were then mapped back to the polished contigs using Minimap2, and mapping quality was assessed with Qualimap v2.2.2a^13^. Contigs with an average read depth below 10-fold were excluded, resulting in a final set of 423 contigs and a total genome size of  173.27 Mb, which were subsequently prepared for scaffolding.

### Hi-C scaffolding

To generate a chromosome-level genome assembly, Hi-C proximity ligation data were utilized. This was achieved through *in-situ* Hi-C sequencing, performed with the EpiTect Hi-C Kit (Qiagen). Sequencing was conducted using the Illumina MiSeq platform, yielding 43.49 million raw reads, equivalent to 13.09 billion bases. After filtration with fastp (v0.23.2), a refined dataset of 27.4 million clean reads, representing 7.4 billion bases, was retained for downstream analysis. These reads were critical for identifying spatially interacting genomic regions, enabling the reconstruction of chromosomal structures. The ARIMA Genomics Hi-C Mapping Pipeline (https://github.com/ArimaGenomics/mapping_pipeline; accessed February 2024) was employed for read alignment and interaction identification. Hi-C reads were aligned using BWA-MEM v0.7.17^14^ in single-end mode, and chimeric or ligation-spanning reads were trimmed to ensure accurate mapping. Mapped reads were filtered based on quality thresholds using Samtools v1.19.2^15^, and PCR duplicates were removed with Picard v3.5.3^16^.

To assemble the genome into candidate chromosomes, YAHS v1.2a.2^17^ was utilized. This assembly process transformed the draft genome into an organized, chromosome-scale structure. The resulting Hi-C maps, generated during assembly, were visualized using HapHic Plot v1.0.3^18^ (Fig. 1).

**Fig. 2:**
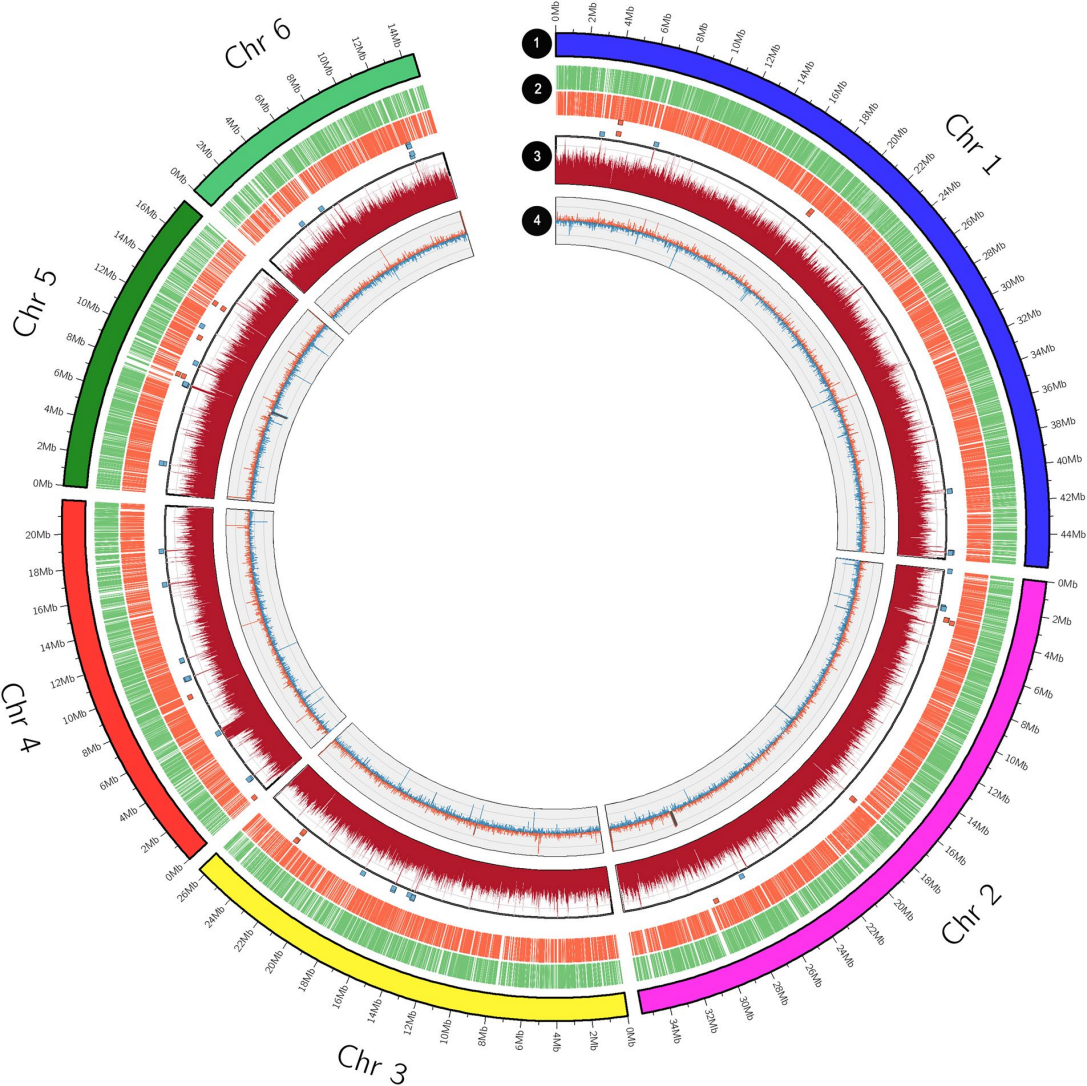
Circos plot illustrating the chromosomal architecture of the *Trichomonas vaginalis* IR-78 genome. Circle 1 shows the six major scaffolds with chromosome lengths indicated in megabases. Circle 2 displays annotated gene sequences in clockwise and anticlockwise orientations. Circle 3 presents the mapping profile of Nanopore long-read data across the chromosomes, with regions of deviated coverage variation highlighted. Circle 4 represents the GC content along the genome.

The final genome assembly of *T. vaginalis* IR-78 was 173.3 Mb in total. Hi-C analysis confirmed the presence of six super-scaffolds of 14, 17, 21, 26, 35, and 45 Mb in size, corresponding to the six chromosomes previously identified in *T. vaginalis*^19^ (Fig. 1). These super-scaffolds collectively anchored 93.5% of the total genome assembly (162.1 Mb). Key metrics of the assembly included a scaffold N50 of 26.4 Mb and a contig N50 of 1.13 Mb, highlighting the assembly’s high contiguity. The average GC content across the genome was calculated to be 32.79% (Table 1, Fig. 2), consistent with previous findings for *T. vaginalis* genomes. This assembly is comparable in size to the *T. vaginalis* G3 strain (assembly name NYU_TvagG3_2, accession GCF_026262505.1)^20^ (Table S1). Despite differences in assembly techniques and sequencing technologies, the IR-78 genome exhibits contiguity comparable to the G3 strain^20^, thus providing a further reliable resource for future comparative genomic studies investigating genomic variation and functionality across *T. vaginalis* and other *Trichomonas* spp. strains.

### Repeat annotation

Tandem repeats in the *T. vaginalis* IR-78 genome were predicted using the Tandem Repeats Finder tool v4.09^21^, which identifies and classifies repeat motifs based on sequence similarity. To annotate other repetitive elements, we used RepeatModeler2 v2.0.5^22^, a *de novo* automated tool designed to construct species-specific transposable element (TE) libraries. These libraries were then used by RepeatMasker v4.1.6 (https://www.repeatmasker.org/) to identify both known and novel TEs in the *T. vaginalis* IR-78 genome^23^. The *T. vaginalis* IR-78 genome is highly enriched for repetitive elements, with a total of 115.3 Mb (66.57% of the genome) classified as repeat sequences. RepeatMasker classified the predicted repetitive sequences as follows: DNA transposons were the most prevalent, comprising approximately 35% of the total genome. Retroelements and long terminal repeat (LTR) elements each represented 0.97%, while simple repeats comprised 0.36%. A significant proportion of the repetitive elements (29.89%) remained unclassified, likely due to the limited research on repetitive sequences in Parabasalia species. Minor proportions of repetitive elements were attributed to rolling-circle elements and short interspersed nuclear elements (SINEs).

As these annotations were generated using automated tools and current repeat databases, which are known to be of limited quality for *T. vaginalis*, the presence of some predicted repeat categories—such as retroelements, LTR elements, rolling-circle elements, and SINEs—should be interpreted with caution. Manual annotation and validation of these sequences are required to confirm their presence and classification, and to rule out potential artifacts arising from automated prediction. As tools for annotation and prediction continue to advance, the classification and robust identification of repetitive elements in *T. vaginalis* genomes are expected to improve, potentially supported by extensive manual curation.

### Annotation of non-coding RNA genes

Homologues of structural RNAs in the *T. vaginalis* IR-78 genome were identified using Infernal v1.1.5^24^ to perform alignments with the Rfam RNA database v14.9^25^ (http://rfam.xfam.org/). The search was carried out with cmscan v1.1.5^24^, revealing 681 high-scoring hits corresponding to structural RNAs, with 460 transfer RNAs (tRNAs) and 165 ribosomal RNAs (rRNAs) (Table 1, Table S2).

### Gene prediction

Protein-coding genes in the *T. vaginalis* IR-78 genome were predicted *de novo* using BRAKER v3.0.8^26–36^. Prior to gene prediction, the assembled genome was soft-masked for repetitive elements using RepeatMasker^22,23^ with the Dfam libraries, in order to reduce spurious gene calls from transposable elements. BRAKER was run in genome-annotation mode using protein homology evidence from the OrthoDB v11 database^37^, which provided conserved orthologous sequences across eukaryotes to guide gene structure inference. Gene models were trained iteratively within BRAKER, and parameters were optimized based on both *ab initio* prediction and external protein alignments. Using this approach, a total of 43,326 protein-coding genes were predicted in the *T. vaginalis* IR-78 genome, with an average gene length of 1,204 base pairs. The genome annotation features are summarized in Table 1 and plotted across the six chromosomes of the *T. vaginalis* IR-78 genome in Fig. 2.

It should be noted that the *de novo* gene prediction workflow applied in this study was not supported by experimental transcriptomic or proteomic evidence, which may render the resulting annotation susceptible to artifacts. Consequently, additional validation will be required to confirm the accuracy of the predicted gene structures. Notably, the automated annotation workflow identified 3961 introns (average length = 232 bp), whereas previous studies have characterized *T. vaginalis* as a largely intron-poor organism^38–40^.

### Functional genome annotation

The functional annotation of the *T. vaginalis* IR-78 genome was carried out using several databases to obtain insights into gene function and biological pathways. The predicted protein sequences of 43,326 genes were analysed with DIAMOND Blastp^35^ (e-value cutoff: 1.0e-3) within the Functional Analysis Module of OmicsBox v3.0.30^41^. Comparisons were made with the databases NR protein database, SWISS-PROT^42^, Gene Ontology (GO)^43^, eggNOG^44^ and KEGG^45^. To complement the BLAST-based functional annotation, InterProScan v5.69-101.0^46^ was used to identify conserved protein domains and motifs using the EMBL-EBI version of InterPro^47^. This allowed a detailed analysis of predicted protein function (Table S3).

The annotation results showed good matches in the above-mentioned functional databases. 42,776 predicted protein sequences had matches in the NR database, 42,663 in RefSeq, and 12,748 in SWISS-PROT. InterProScan identified functional domains in 31,664 predicted protein sequences, which corresponds to 73.1% of the predicted proteome. Assignment to Gene Ontology (GO) terms revealed that 21,038 predicted protein sequences (48.5%) could be assigned to specific biological processes, molecular functions and cellular components. 10,015 sequences (23.1%) were associated with enzyme code (EC) annotations, linking these predicted proteins to specific enzymatic activities. Further pathway analysis using eggNOG revealed that 22,149 sequences (51.1%) were associated with orthologous groups (OG) and functional categories. The KEGG database identified 414 metabolic and signalling pathways, encompassing 9,488 sequences associated with these pathways. Reactome analysis^48^ identified a further 2,274 metabolic pathways, encompassing 1,472 protein sequences.

**Fig. 3:**
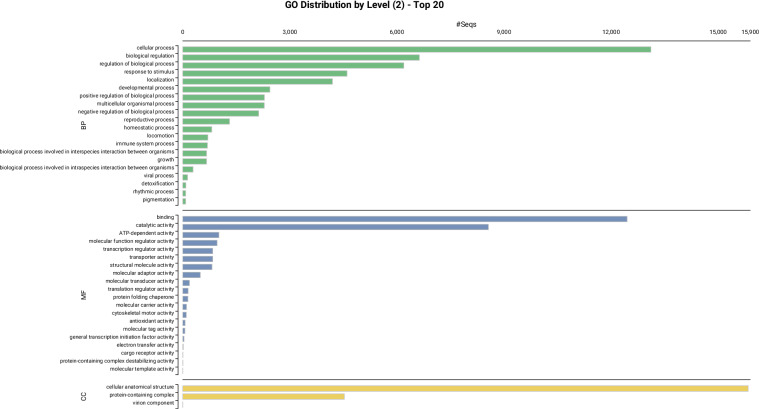
Gene Ontology (GO) categorization of functionally annotated genes in the *Trichomonas vaginalis* IR-78 genome. The chart summarizes the distribution of gene functions across biological process (green), molecular function (blue), and cellular component (yellow) domains, based on GO term assignments.

Gene Ontology classification of the functionally annotated genes revealed that they are distributed across three primary GO domains: biological process, molecular function and cellular component. Of the annotated genes, 32% (n = 13,899) were associated with ‘biological process’, 40.6% (n = 17,612) with ‘Molecular Function’ and 41% (n = 17,783) with ‘cellular component’ (Fig. 3). In the ‘Biological Process’ category, the GO terms at level 2 ‘cellular process’ (39%, n = 16,064) and ‘metabolic process’ (30%, n = 13,115) were the most common classes. In the ‘Cellular Component’, the predominant GO terms were ‘cellular anatomical entity’ (36.5%, n = 15,840), which represents genes associated with structural and organelle functions, and ‘protein complex’ (10.4%, n = 4,529). The most abundant GO classes in the ‘molecular function’ class included ‘binding’ (28.7%, n = 12,451) and ‘catalytic activity’ (19.7%, n = 8,565), indicating a significant presence of enzymes that drive the organism’s biochemical processes (Fig. 3 and Tables S3, S4).

## Data Records

All raw data of the whole genome have been deposited at the National Center for Biotechnology Information (NCBI) under the SRA accession number SRP589323^49^. The completed genome assembly is available in GenBank under the accession number GCA_052324655.1^50^. The corresponding annotation data have been deposited on Zenodo and can be accessed via the following link: https://zenodo.org/records/16924891^51^.

## Technical Validation

### Quality assessment of the genome assembly

To validate the genome assembly, QUAST^52^ was utilised to report assembly contiguity metrics. Additionally, we calculated the percentage of long reads mapping to the final assembly. For this, nanopore data were mapped using minimap2, and mapping quality was assessed with Qualimap^13^. Genome completeness was evaluated using two approaches. First, we used BUSCO v5.5.0^53^ with the eukaryota_odb10 dataset, which assesses genome completeness by searching for conserved single-copy orthologs across eukaryotic species. The analysis identified 127 complete BUSCOs, corresponding to a completeness score of 49.8%, consistent with ranges reported for other Parabasalia genomes^54,55^. BUSCO’s focus on evolutionarily conserved orthologs makes it a reliable benchmark for cross-species comparisons for genome integrity. However, the relatively low BUSCO score reflects the absence of representative genes for highly divergent protists in the reference set, rather than a true deficiency of the assembly. Currently, no universally appropriate benchmark exists for genome completeness in such evolutionary distant lineages.

Additionally, we employed OMARK v0.3.0^56^, a hierarchical orthology analysis tool designed to detect conserved Hierarchical Orthologous Groups (HOGs) across eukaryotic lineages. Using the Eukaryota ancestral clade, OMARK estimated a completeness score of 71.26%, identifying 709 of the 995 conserved HOGs. Among these, 32.96% were classified as single-copy, 38.29% as duplicate, and 28.74% as missing. Of the 43,325 predicted proteins, 14,897 (34.38%) showed consistent lineage placement, reflecting a substantial alignment with established phylogenetic expectations.

### Chromosome synteny

**Fig. 4:**
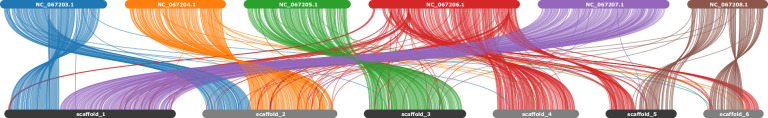
Synteny analysis between the *Trichomonas vaginalis* IR-78 sequenced in this study (bottom) and the reference genome of *T. vaginalis* G3 strain (top). Conserved genomic regions are displayed as colored links, illustrating collinear blocks and highlighting the extent of genome conservation and rearrangement between the two strains.

To evaluate the structural completeness and accuracy of the genome assembly of *T. vaginalis* strain IR-78 (ATCC 50138), we conducted a synteny analysis against the reference genome of strain G3 (NC_067203.1 – NC_067208.1)^20,57^. Chromosome synteny was generated using MCScanX v1.0.0^58^ with default parameters and visualized with SynVisio (no version)^59^. The resulting plot (Fig. 4) shows a high degree of collinearity and conservation between the two strains, as expected for intraspecies comparisons. However, several ribbons span across different chromosomes may suggest either genuine structural variations (e.g., inversions or rearrangements) or assembly-related fragmentation in the IR-78 genome.

## Supplementary information

Supplementary Tables 1 - 4.

## Data Availability

The datasets generated and analyzed in this study are publicly available. The raw sequencing data have been deposited at NCBI under the SRA accession number SRP589323, BioProject accession number PRJNA1271105, and BioSample accession number SAMN48852979. The completed genome assembly is available in GenBank under the accession number GCA_052324655.1. The corresponding genome annotation is accessible via Zenodo at https://zenodo.org/records/16924891.

## Code availability

All software and tools in this study were used with their default parameters, unless otherwise stated.
